# Impact of Osteopathic Treatment on Pain in Adult Patients with Cystic Fibrosis – A Pilot Randomized Controlled Study

**DOI:** 10.1371/journal.pone.0102465

**Published:** 2014-07-16

**Authors:** Dominique Hubert, Lucile Soubeiran, Fabrice Gourmelon, Dominique Grenet, Raphaël Serreau, Elodie Perrodeau, Rafael Zegarra-Parodi, Isabelle Boutron

**Affiliations:** 1 Adult CF Center and Pulmonology Department, Hôpital Cochin APHP, Paris, France; 2 Université Paris Descartes, Sorbonne Paris Cité, Paris, France; 3 Clinical Research Department, Hôpital Cochin APHP, Paris, France; 4 Adult CF Center and Pulmonology Department, Hôpital Foch, Suresnes, France; 5 Clinical Epidemiology Center, Hôpital Hôtel Dieu APHP, Paris, France; 6 A.T. Still Research Institute, A.T. Still University, Kirksville, Missouri, United States of America; University of Ottawa, Canada

## Abstract

**Background:**

Pain is a common complication in patients with cystic fibrosis (CF) and is associated with shorter survival. We evaluated the impact of osteopathic manipulative treatment (OMT) on pain in adults with CF.

**Methods:**

A pilot multicenter randomized controlled trial was conducted with three parallel arms: OMT (group A, 16 patients), sham OMT (sham treatment, group B, 8 patients) and no treatment (group C, 8 patients). Medical investigators and patients were double-blind to treatment for groups A and B, who received OMT or sham OMT monthly for 6 months. Pain was rated as a composite of its intensity and duration over the previous month. The evolution of chest/back pain after 6 months was compared between group A and groups B+C combined (control group). The evolution of cervical pain, headache and quality of life (QOL) were similarly evaluated.

**Results:**

There was no statistically significant difference between the treatment and control groups in the decrease of chest/back pain (difference = −2.20 IC95% [−4.81; 0.42], p = 0.098); also, group A did not differ from group B. However, chest/back pain decreased more in groups A (p = 0.002) and B (p = 0.006) than in group C. Cervical pain, headache and QOL scores did not differ between the treatment and control groups.

**Conclusion:**

This pilot study demonstrated the feasibility of evaluating the efficacy of OMT to treat the pain of patients with CF. The lack of difference between the group treated with OMT and the control group may be due to the small number of patients included in this trial, which also precludes any definitive conclusion about the greater decrease of pain in patients receiving OMT or sham OMT than in those with no intervention.

**Trial Registration:**

ClinicalTrials.gov NCT01293019

## Introduction

Cystic Fibrosis (CF) is an inherited multi systemic autosomal recessive disease with high morbidity and mortality, involving primarily the lungs and gastrointestinal tract [1]. The daily routine of a CF patient generally requires long-term home care, including oral and inhaled antibiotics, pancreatic enzyme replacement, mucolytic agents, vitamin supplements, and daily physiotherapy. In addition, these patients often require home or in-hospital intravenous antibiotic therapy during pulmonary exacerbations. Significant advances in the management of respiratory infection and pancreatic insufficiency, coupled with better quality of care by specialist multidisciplinary teams, have resulted in a significant improvement in the median survival age, which approaches 40 years [2]–[3]. Until recently, the majority of patients with CF were paediatric. Today, many reach adulthood and, in many countries there are now as many adults as children, if not more [2]–[3]. Consequently, greater attention is being paid to patients’ quality of life (QOL). CF patients frequently complain of pain, mainly back pain, chest pain and headache [4]–[11]. Although pain in adults with CF is associated with lower QOL [5]–[6], [11], increased risk of exacerbation [6] and decreased survival [6], [12], it is often neglected by the treating physicians [5], [8]. In addition to drug treatment, many patients with CF use non-drug treatments and complementary and alternative medicine (CAM) [5]–[6], [8], [13]–[15] including osteopathic manipulative treatment (OMT). However, this type of treatment has never been evaluated in CF.

The aim of this pilot study was to demonstrate the feasibility of evaluating the efficacy of OMT to treat the pain of adult patients with CF.

## Materials and Methods

The protocol for this trial and supporting CONSORT checklist are available as supporting information; see Checklist S1 and Protocol S1.

### Ethic Statement

The study was approved by our Institutional Review Committee (Comité de Protection des Personnes “Ile de France II” # 2009-A00359-48) and all subjects provided written informed consent. The study was registered at ClinicalTrials.gov #NCT01293019 on February 9, 2011 (http://clinicaltrials.gov/ct2/show/NCT01293019). The delay in registering the study was due to a change in the statistician responsible for the study. The authors confirm that all ongoing and related trials for this intervention are registered.

### Study Design

This pilot study was a three-arm, parallel group, randomized controlled trial.

### Setting and Participants

The study was carried out in two adult CF centers: Cochin University Hospital, Paris, France; and Foch Hospital, Suresnes, France.

The inclusion criteria were age 18 or older, diagnosis of CF (positive sweat test and/or two CFTR disease-causing mutations) and cervical, back or chest pain, rated 2 or higher on a 10-point visual analog scale (VAS) [16] or requiring painkillers during the previous month. The exclusion criteria were treatment with OMT or other forms of spinal manipulation in the past three months, pregnancy, lung transplantation, being on a waiting list for lung transplantation, and participation in another clinical trial.

### Randomization and Treatment

The patients were randomly assigned to one of the three groups: OMT (experimental treatment) for 16 patients (group A), sham OMT (sham treatment) for 8 patients (group B) and no additional treatment (usual care) for 8 patients (group C). Randomization was performed according to a computer-generated list with blocks of fixed size, stratified by center. In each block of four, there were two arms A, one arm B and one arm C. The list was prepared and maintained by an independent statistician at an independent clinical trial unit. The investigators did not have access to the randomization list, and allocation was concealed through an internet-based system.

For cases receiving experimental treatment or sham OMT, medical investigators, patients and outcome assessors were blinded, and only the osteopathic practitioner knew the allocation to the treatment arm. However, blinding was not feasible for cases receiving usual care without OMT.

### Intervention and Comparator

The experimental treatment was OMT. The intervention consisted of six monthly sessions of OMT. All OMT was provided by a single osteopathic practitioner (LS) trained according to international standards [17] and who had four years of experience in treating CF patients.

A standardized treatment plan for diagnosed somatic dysfunction was used [18]–[20]. Each osteopathic visit lasted 1 hour and consisted of three periods for both groups: (1) interview focusing on pain location, (2) full osteopathic examination [21] (Table S1), (3) intervention consisting of either OMT or light touch sham OMT according to the randomization arm (Table S2, Table S3, Table S4). (See the supplementary Methods section for more details).

The control group was divided into two groups. One group was treated according to usual care and received no OMT. Patients in the sham OMT group received light touch at the skull and at the sacrum, a sham osteopathic procedure used in previous trials [22]–[23], to ensure that manual contact time was similar in the experimental and sham OMT groups.

### Outcomes

The primary efficacy endpoint was a composite outcome reflecting the intensity of pain and the number of painful days over the previous month evaluated after 6 months. For each patient, we calculated the mean value of the two following variables: (1) chest and back pain over the previous month (VAS from 0 to 10) [15] and (2) [the number of days with chest and back pain in the previous month/number of days in the previous month] ×10 (thus a score from 0 to 10).

Secondary endpoints included:

Pain involving the neck and trapezius at 6 months evaluated as the mean value of: neck and trapezius pain over the previous month (VAS from 0 to 10) and [the number of days with neck and trapezius pain in the previous month/number of days in the previous month]×10 (score from 0 to 10).
Headache at 6 months evaluated as the mean value of: headache over the previous month (VAS from 0 to 10) and [the number of days with headache in the previous month/number of days in the previous month]×10 (score from 0 to 10).
QOL scores at 6 months with validated French-language versions of the questionnaire (Cystic Fibrosis Questionnaire (CFQ) 14+) [24]. Four domains were studied: physical functioning; energy/well-being; body image; and respiratory symptoms.
The need for analgesics.

At the end of the study, all patients completed a questionnaire to collect data about their interest in the study and their satisfaction with the treatment received.

### Data Collection

At the base-line visit, we collected demographic data and information about the characteristics of the patient’s CF, including respiratory function evaluated by measuring forced expiratory volume in one second (FEV1), and nutritional status evaluated with the body mass index (BMI). All patients were evaluated at the base-line visit (M0), after 3 months (M3) and after 6 months (M6) at the end of the study. Upon entry into the study, the participants were given a diary in which they recorded their pain symptoms(location and intensity) daily at baseline and during the trial; this diary was kept at home and brought for each study visit, and returned to the investigators at the end of the study. The CF physicians (DH or DG) checked the patient’s diary and recorded the patient’s pain after the patient had self-reported his pain on a VAS (mean value and number of painful days in the previous month), collected the CFQ 14+, listed concomitant treatment including analgesics, and adverse events. The osteopathic practitioner (LS) performed osteopathic tests for all patients.

### Sample Size

We planned to include 32 subjects (16 in the OMT group, 8 in the sham OMT group and 8 in the usual care group). Because this study was a pilot study in a rare disease, there was no formal calculation of sample size; nevertheless, this sample was expected to be sufficient to identify a difference of one standard deviation between the mean VAS for pain in the OMT group versus the two other groups with 1−β = 80% and α = 5%.

### Statistical Analyses

All randomized patients were analyzed in their allocated group whatever the treatment received.

Baseline data are described using means and standard deviations (SD) for quantitative variables, and medians and interquartile ranges (IQR) for variables with asymmetric distributions. For qualitative variables, frequencies and percentages were computed.

Primary comparisons were between the OMT group (group A) and the sham OMT group+no additional treatment group pooled (group B+C).

The difference for the primary outcome between the two groups was evaluated at 6 months using a linear mixed longitudinal model estimating the difference in change from baseline between the 2 groups (coefficient for time × group interaction) and taking into account the correlation for data of a same patient (random effect). This difference was tested using the Student’s *t*-test at a 5% level in order to test the non nullity of the coefficient of interest in the model.

Pairwise comparisons between all three groups were also performed at a 1.67% level to take into account the multiplicity induced by these three comparisons. To check consistency with the parametric analysis, Wilcoxon’s tests with significance set at 5% were used for a non-parametric comparison of the difference between the two groups in the distribution of changes from baseline to month 6.

Final composite outcomes (M6) for neck and trapezius pain, headache and QOL scores were analyzed with the same strategy as the primary outcome.

The need for analgesics was analyzed using a mixed logistic regression model with a random intercept at the individual patient level.

The nlme package [25] from R software version 2.14 [26] was used for statistical analyses.

## Results

**Figure 1:**
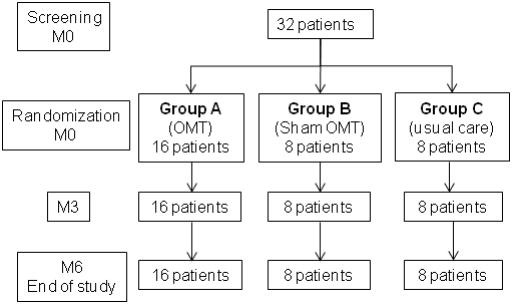
Flow chart.

Thirty-two patients were included between November 2009 and October 2010; 16 patients were randomized to osteopathic manipulative treatment (group A), eight patients to sham OMT (group B) and eight patients to usual care (group C). No patient dropped out of the study after randomization, and all 32 patients attended all the visits planned in the study until month 6. The last study visit took place on April 2011. Results for all 32 patients were included in the analysis (Figure 1).

Demographic and clinical factors are reported in Table 1.

**Table 1:** Baseline characteristics of the study participants.

Characteristics	Osteopathic treatment (Group A) N = 16	Sham and usual care (Groups B+C) N = 16
**Age** (yr) – mean ± SD	31±6	37±11
**Sex**		
Male – n (%)	1 (6.2)	5 (31.2)
Female – n (%)	15 (93.8)	11 (68.8)
**FEV1 (% pred) - mean ± SD**	53.9±22.6	46.8±18.7
**BMI** (kg/m^2^) -mean ± SD	21.6±3.8	22.1±4.0
**Chest/Back pain -** med, [Q1;Q3]		
VAS (range 0–10)	5.5 [5]; [7]	5.0 [4]; [7]
Days in the previous month (range 0–30)	30 [24.2;30]	30 [30;30]
Composite outcome(range 0–10)	7.5 [6.2;8.1]	7.5 [6.9;8.1]
**Neck/Trapezius pain -** med, [Q1;Q3]		
VAS (range 0–10)	1.5 [0;5]	3.0 [0;5]
Days in the previous month (range 0–30)	2 [0;27]	30 [0;30]
Composite outcome(range 0–10)	1.8 [0;6.6]	6.5 [0;7.5]
**Headache -** med, [Q1;Q3]		
VAS (range 0–10)	3.0 [0;5.2]	0 [0;4.2]
Days in the previous month (range 0–30)	1.5 [0;30]	0 [0;3]
Composite outcome(range 0–10)	2.5 [0;7.1]	0 [0;1.2]
**QOL scores -** med, [Q1;Q3] (range 0–100)		
Physical functioning	50 [32;56.2]	40 [21;57.2]
Energy/well-being	50 [23;58]	37.5 [25;52]
Body image	67 [56;78]	67 [44;78]
Respiratory symptoms	52 [51;63.2]	55 [32;76]

**Figure 2:**
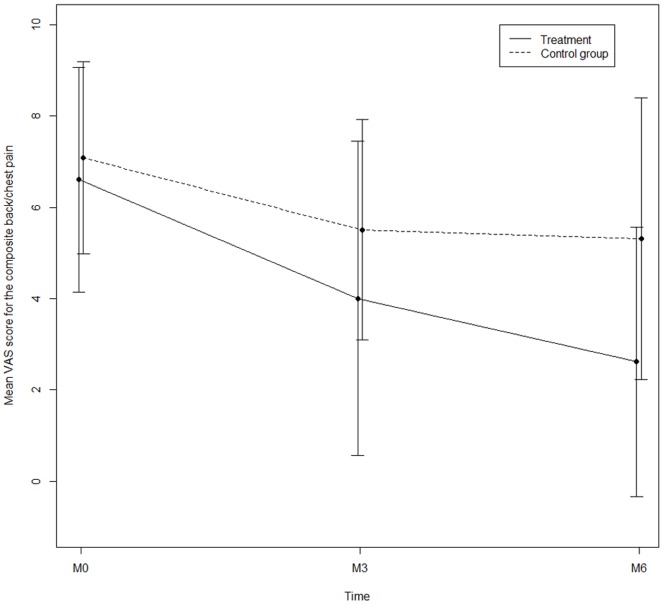
Composite chest/back pain at inclusion (M0), after 3 months (M3) and at the end of the study (M6) in the OMT group (A) and control group (B+C). VAS: visual analog scale.

Figure 2 and Table 2 show the changes in the primary outcome measure (composite chest/back pain) between the beginning and the end of the study in the experimental treatment and control groups. There was no statistically significant difference in the decrease of chest/back pain between the treatment group and the control group (difference = −2.20 IC95% [−4.81; 0.42], p = 0.098).

**Table 2:** Change in the primary outcome measure (composite chest/back pain) between the first and the final visit in the three treatment groups and comparisons between groups.

	Composite chest/back pain in the OMT group (Group A) N = 16 Mean(SD)	Composite chest/back pain in the sham OMT group (Group B) N = 8 Mean(SD)	Composite chest/back pain in the usual care group (Group C) N = 8 Mean(SD)	Composite chest/back pain in the sham OMT or usual care group (Group B+C) N = 16 Mean(SD)	Mean change difference in composite chest/back pain between Group A and Group B+C [95%CI]	Mean change difference in composite chest/back pain between Group A and Group B [95% CI]	Mean change difference in composite chest/back pain between Group A and Group C [95% CI]	Mean change difference in composite chest/back pain between Group B and Group C [95% CI]
M0	6.60 (2.47)	7.94 (1.02)	6.23 (2.61)	7.08 (2.11)				
M3	4.00(3.44)	5.40 (2.07)	5.62 (2.86)	5.51 (2.41)	−1.08 [−3.34; 1.18]	−0.13 [−2.82; 2.57]	−2.09 [−4.79; 0.62]	−1.96 [−5.05;1.14]
M6	2.61 (2.95)	3.77 (3.65)	6.85 (1.26)	5.31 (2.08)	−2.20 [−4.81;0.42]; p = 0.1	0.19 [−2.70;3.08]; p = 0.9	−4.62 [−7.52; −1.72]; p = 0.002	−4.81 [−8.15; −1.47]; p = 0.006

SD: standard deviation.

95% CI: 95% confidence interval.

**Figure 3:**
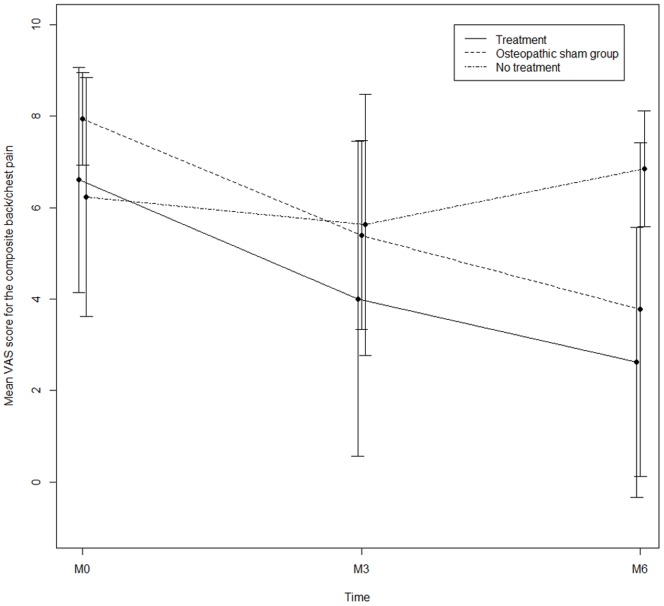
Composite chest/back pain at inclusion (M0), after 3 months (M3) and at the end of the study (M6) in the OMT group (A), the OMT sham group (B) and the group with only usual treatment (C). VAS: visual analog scale.

We performed prespecified ancillary analyses for the primary outcome (composite chest/back pain) in the three groups, differentiating between the sham OMT (group B) and no treatment (group C) groups (Figure 3 and Table 2). The primary outcome measure did not differ between patients who had received sham OMT and OMT (difference = 0.19 [−2.70 to 3.08], p = 0.9). However, chest/back pain was lower in the OMT (difference = −4.62 [−7.52 to −1.72], p = 0.002) and sham (difference: −4.81 [−8.15 to −1.47], p = 0.006) groups than in the usual care group.

**Figure 4:**
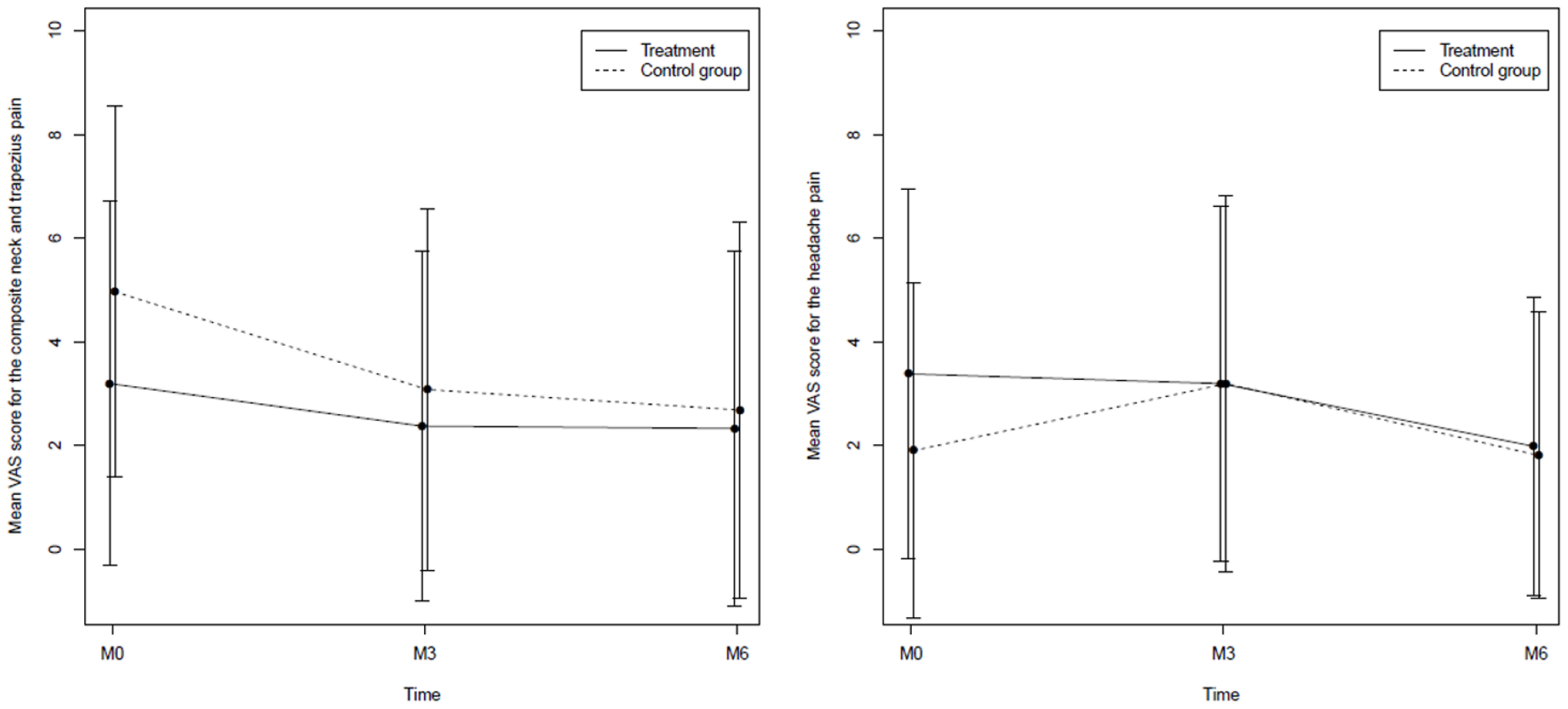
Composite neck/trapezius pain and headache at inclusion (M0), after 3 months (M3) and at the end of the study (M6) in the OMT group (A) and control group (B+C). VAS: visual analog scale.

No differences between the treatment group and the control group (group B+C) were found for the secondary outcome measures cervical pain, headache (Figure 4 and Table 3), or QOL.

**Table 3:** Mean neck/trapezius pain and headache between the first and the final visit for the treatment group and the control group.

	Composite neck/trapezius pain		Headache	
	OMT group (Group A) N = 16	sham OMT or usual care group(Group B+C) N = 16	OMT group (Group A) N = 16	Sham OMT or usual care group(Group B+C) N = 16
Mean (standard deviation)				
M0	3.20 (3.51)	4.97 (3.58)	3.39 (3.57)	1.91 (3.24)
M3	2.38 (3.37)	3.08 (3.48)	3.20 (3.42)	3.20 (3.63)
M6	2.33 (3.43)	2.69 (3.63)#	1.99 (2.88)	1.81 (2.76)##

# The difference (95% confidence interval) between Groups A and B+C was not significant: 1.51 (−0.97; 3.98)– p = 0.228.

## The difference (95% confidence interval) between Groups A and B+C was not significant: −1.30 (−3.27; 0.66)– p = 0.190.

When taking into account the mean VAS score and the mean number of days in pain in all arms of the study, the results were similar (Table S5). The difference of change from baseline for chest/back pain between the experimental group treated with OMT and the control group was −1.96 [−4.12; 0.22] for mean VAS [CI 95%]), p = 0.076.

All non-parametric analyses gave consistent results (not shown).

The need for analgesics did not differ significantly between groups (Table 4). Although it remained stable for patients with usual care and decreased for patients with OMT or sham treatment, the numbers of patients were too small for conclusions to be drawn.

**Table 4:** Need for analgesics in the OMT group, the sham OMT group and the group with usual care.

Number of patients using analgesics (%)			
	OMT group (Group A) N = 16	sham OMT group (Group B) N = 8	Usual care group (Group C) N = 8
M0	10 (62)	5 (62)	4 (50)
M3	7 (44)	3 (37)	3 (37)
M6	4 (25)	2 (25)	4 (50)

No harm or unintended effect was reported due to treatment.

Twenty-six patients (81%) indicated that they had been interested in the study and six (19%) that they had been slightly interested. Fifteen of the 16 patients in the treatment arm were very satisfied (94%) and one was reasonably satisfied (6%); five of the eight patients in the sham group were very satisfied (62%), two were reasonably satisfied (25%) and one patient (12%) was not satisfied.

## Discussion

Our pilot study is the first to demonstrate that it is feasible to evaluate OMT against the pain affecting CF patients. However, it did not show any difference in the decrease of pain between the group treated with OMT and the control group, though chest/back pain decreased more in patients receiving OMT and sham OMT than in those with no intervention. Our study was therefore not powered to be able to draw firm conclusions on the efficacy of OMT against the pain of patients with CF.

A sufficient number of patients were rapidly recruited (over 12 months rather than over the 18 months initially planned for) and none of the 32 patients dropped out of the study. This reflects the significant expectations that CF patients have for complementary medicine for pain relief. In a survey in French CF patients, 326 of 714 respondents (46%) had used CAM and 38% had experience of OMT [14]. In a survey on pain including 239 Italian adults with CF, 22.2% used non-pharmacological remedies including massage [8]. Hayes reported that 45% of 83 adult CF patients practiced chiropractic, yoga or massage for pain relief [6]. In a study of 97 pediatric CF patients, 77% used some method of CAM including manipulative techniques [15]. Only one study has evaluated the effects of manual mobilization techniques and massage therapy on pain: including 105 adults with CF, it reported positive results [27]; however, the study had limitations, and in particular there was no control group such that it could not reveal any placebo effect.

In our study, it is noteworthy that the majority of our patients were women. Indeed, in CF there is a female disadvantage in terms of survival and morbidity, called the “CF gender gap” [28], which might be due to the female sex hormone estrogen. Nevertheless, it is unclear from the literature whether women with CF are in greater pain than men with CF.

As complementary medical techniques become more mainstream, we anticipate that more adults with CF will attempt alternative therapies for pain relief. Our study is the first to assess a treatment of this type in CF patients. We chose to evaluate the effects of OMT on chest/back pain because pain at these sites is prevalent among adults with CF, affecting 15–72% of CF patients [4]. While some patients seek relief from their pain with osteopathic practitioners (but no more than a few times per year), this treatment is not currently approved in CF and is not covered by public health insurance in France. We opted for a monthly treatment regimen as the optimal OMT regimen is not known. Indeed, there are no existing studies on dose-response effects of OMT, nor are there any recommendations or studies on the optimal frequency of OMT. The only available data are descriptive and refer to the frequency of treatment in current practice [29]. Our choice for monthly treatments relied on expert opinions. Regarding the control group, we divided it into patients receiving usual care and those receiving sham OMT, so as to evaluate the feasibility of using sham OMT. Because sham treatment is difficult to implement in clinical trials of OMT [30], we chose to use light touch at the skull and at the sacrum which has been used as a placebo method in trials evaluating OMT [22]–[23]. Cranial palpation, equivalent to light touch, has been proposed as a sham OMT treatment option [31]. Although the results of a systematic review on the effects of craniosacral therapy suggest improvements on general wellbeing/quality of life and pain, the moderate quality of current studies and the scarcity of available data prevent any definitive conclusion regarding the clinical effectiveness of this type of OMT [32].

We found no statistically significant difference in the decrease of chest/back pain between patients treated with OMT and the control group. There was no statistically significant difference between patients receiving the OMT intervention treatment and those receiving the OMT sham treatment. However, chest/back pain decreased significantly more in patients receiving the OMT intervention treatment and in those receiving the osteopathic sham treatment than in patients only receiving usual routine care. There was also no difference between the intervention group and the control group with respect to neck/trapezius pain, headache, QOL scores or the need for analgesics. It has been reported that alternative medicine may be an especially successful placebo-generating health care system [33]–[34]. Clinical outcomes of OMT result from both OMT-specific and placebo mechanisms (e.g., importance of patient characteristics, practitioner characteristics, patient-practitioner interaction, length of patient encounter) [35].

A systematic review and meta-analysis of randomized, controlled trials assessed the effect of OMT on chronic low back pain: it concluded that OMT reduced low back pain significantly more than expected from placebo effects, and that the beneficial effects persisted for at least three months [36]. In a trial comparing standard medical therapies and OMT over a 12-week period in 155 patients, there was no statistically significant difference between the two groups in any of the primary outcome measures, but the OMT group required significantly less analgesic medication and used less physical therapy [37]. However, this study was not blinded, making it is difficult to distinguish the treatment effect from the placebo effect. In another trial, 91 subjects with chronic low back pain were randomized to OMT, sham manipulation, or a no-intervention control group [38]. As in our study, both the patients who received OMT and those who received the sham manipulation reported greater improvement in back pain than the no-intervention control subjects. It is unclear whether the benefits of OMT could be attributed to the manipulative techniques themselves or whether they were related to other non-specific aspects of OMT [38].

In conclusion, our pilot study shows that the assessment of the effects of OMT on chest and back pain in adults with CF is feasible. Patients have substantial expectations and hopes for such studies. The lack of any significant difference between the intervention OMT group and the control group might be related to the small number of patients, which also precludes any definitive conclusion about the greater decrease of pain in patients receiving OMT or sham OMT than in those with no intervention. This may raise the question of a placebo effect and highlights the importance of psychological factors and human relationships in the treatment of pain. Nevertheless, the results need to be interpreted with caution, due to the small number of patients in our pilot study and the multiplicity of the tests. Larger and more powerful studies are needed to determine whether OMT could be beneficial for CF patients who suffer chest and back pain.

## Supporting Information

Table S1 **Criteria for clinical decision about the severity of somatic dysfunction according to the three categories of tests.**
(DOCX)

Table S2 **Different techniques of the Osteopathic Manipulative Treatment.**
(DOCX)

Table S3 **Intervention used in the two** **OMT groups.**
(DOCX)

Table S4 **Position of the patient and of the practitioner during the different techniques.**
(DOCX)

Table S5 **Change in pain intensity and days in pain between the experimental OMT group (A) and the control group (B+C).**
(DOCX)

Checklist S1 **CONSORT checklist.**
(DOC)

Protocol S1 **Trial protocol.**
(DOCX)

Methods S1 **Supplementary methods.**
(DOCX)
