# Supplementary material for: Impact of Osteopathic Treatment on Pain in Adult Patients with Cystic Fibrosis – A Pilot Randomized Controlled Study
Source: PLoS One. 2014 Jul 16;9(7):e102465. doi: 10.1371/journal.pone.0102465 (PMC4100932; doi:10.1371/journal.pone.0102465)
Supplement: Table S1 — Criteria for clinical decision about the severity of somatic dysfunction according to the three categories of tests. (DOCX) [file pone.0102465.s001.docx]

**Table S1.** Criteria for clinical decision about the severity of somatic dysfunction according to the three categories of tests

| **Category of osteopathic tests** | **Anatomical areas/**  **Palpation over** | **Absence of somatic dysfunction (severity scored as 0)** | **Mild somatic dysfunction**  **(severity scored as 1)** | **Moderate somatic dysfunction**  **(severity scored as 2)** | **Severe somatic dysfunction**  **(severity scored as 3)** |
| --- | --- | --- | --- | --- | --- |
| Cranial | Bony landmarks  Cranial sutures | No clinical signs associated with somatic dysfunction | 1-2 clinical signs associated with somatic dysfunction | Restriction of mobility and at least 2 signs associated with somatic dysfunction | All 4 clinical signs associated with somatic dysfunction |
| Visceral | The different organs |  |  |  |  |
| Musculoskeletal | Cervical  T1-T4  T5-T9  T10-T12  Lumbar  Pelvis/Sacrum  Pelvis/Innominate  Lower extremities (right and left)  Upper extremities (right and left)  Ribs |  |  |  |  |

T: Thoracic
