# Supplementary material for: Impact of Osteopathic Treatment on Pain in Adult Patients with Cystic Fibrosis – A Pilot Randomized Controlled Study
Source: PLoS One. 2014 Jul 16;9(7):e102465. doi: 10.1371/journal.pone.0102465 (PMC4100932; doi:10.1371/journal.pone.0102465)
Supplement: Table S2 — Different techniques of the Osteopathic Manipulative Treatment. (DOCX) [file pone.0102465.s002.docx]

**Table S2.** Different techniques of the Osteopathic Manipulative Treatment

| **Cranial techniques** | **Muscle techniques** | **Structural techniques** | **Visceral techniques** |
| --- | --- | --- | --- |
| - Gentle manual force over bony landmarks  - Gentle manual force over the venous system | - Inhibition  - Stretching  - Rhythmic soft tissue | - Joint mobilization  - Joint manipulation | - Myofascial technique of the thoracic inlet, lung cylinders and mediastinum  - Gentle manual force over the sphincters and the pleural dome  - Stretching of the diaphragm  - Light rhythmic pressure of the liver  - Stretching technique in projection of the root of the mesentery, the ties of the caecum and colon, the colic angles |
