# Supplementary material for: Impact of Osteopathic Treatment on Pain in Adult Patients with Cystic Fibrosis – A Pilot Randomized Controlled Study
Source: PLoS One. 2014 Jul 16;9(7):e102465. doi: 10.1371/journal.pone.0102465 (PMC4100932; doi:10.1371/journal.pone.0102465)
Supplement: Table S3 — Intervention used in the two OMT groups. (DOCX) [file pone.0102465.s003.docx]

**Table S3.** Intervention used in the two OMT groups

|  | **OMT (experimental treatment)** | **Sham OMT (sham group)** |
| --- | --- | --- |
| Osteopathic manipulative treatment plan | Chest/Back pain  Structural, visceral or cranial SD, in conjunction with the theoretical chest SD | None |
| Techniques | Structural  Visceral  Cranial | Light touch to the skull  Light touch to the sacrum |
