# Supplementary material for: Impact of Osteopathic Treatment on Pain in Adult Patients with Cystic Fibrosis – A Pilot Randomized Controlled Study
Source: PLoS One. 2014 Jul 16;9(7):e102465. doi: 10.1371/journal.pone.0102465 (PMC4100932; doi:10.1371/journal.pone.0102465)
Supplement: Table S4 — Position of the patient and of the practitioner during the different techniques. (DOCX) [file pone.0102465.s004.docx]

**Table S4*.*** Position of the patient and of the practitioner during the different techniques

| **Anatomical areas** | **Techniques used** | **Patient’s position** | **Practitioner’s position** |
| --- | --- | --- | --- |
| Cranial | Gentle manual force over the bony landmarks  Gentle manual force over the venous system | Supine | Sitting at the head |
| Cervical | Inhibition of the suboccipital muscles  Spinal mobilization | Supine | Sitting at the head or standing at the head |
| T1-T4 | Soft tissues techniques  Stretching of the neck muscles  Spinal manipulation | Prone  Supine | Standing at the side  Standing at the head |
|  | Compression or stretching on the inspiratory accessory muscles  Spinal manipulation | Supine or prone | Sitting at the head |
| T10-T12 | Spinal mobilization  Spinal manipulation | Seated  Supine or prone | Standing behind  Standing aside |
| Lumbar | Lumbar soft tissue  Spinal mobilization  Spinal manipulation | Prone  Lateral supine | Standing aside |
| Pelvis/sacrum | Joint mobilization  Joint manipulation | Lateral supine | Standing aside |
| Pelvis innominate | Joint manipulation | Supine | Standing aside |
| Lower right or left extremity | Mobilization of the lower limb joints  Stretching of the muscles  Soft tissues techniques | Supine | Standing aside  Standing or sitting |
| Upper right or left extremity | Mobilization of the upper limb joints  Stretching of the muscles  Soft tissues techniques | Supine | Standing aside |
| Ribs | Spinal manipulation | Supine | Standing aside |
| Visceral | Gentle manual force on the organ  Myofascial technique on the organ  Stretching technique in projection | Supine | Standing aside |

T: Thoracic
