# Supplementary material for: Impact of Osteopathic Treatment on Pain in Adult Patients with Cystic Fibrosis – A Pilot Randomized Controlled Study
Source: PLoS One. 2014 Jul 16;9(7):e102465. doi: 10.1371/journal.pone.0102465 (PMC4100932; doi:10.1371/journal.pone.0102465)
Supplement: Table S5 — Change in pain intensity and days in pain between the experimental OMT group (A) and the control group (B+C). (DOCX) [file pone.0102465.s005.docx]

**Table S5 .** Change in pain intensity and days in pain between the experimental OMT group (A) and the control group (B+C)

|  | | Group A | | Group B+C | |
| --- | --- | --- | --- | --- | --- |
|  |  | M0 | M6 | M0 | M6 |
| **Chest / back pain** mean (SD) | Pain intensity (VAS) | 5.38 (1.78) | 1.94 (2.46) | 5.06 (2.14) | 3.56 (2.48) |
|  | Days over the previous month | 24 (12) | 10 (13) | 27 (8) | 21 (14) |
| **Neck pain**  mean (SD) | Pain intensity (VAS) | 2.81 (3.25) | 2.19 (3.04) | 3.06 (2.79) | 1.63 (2.45) |
|  | Days over the previous month | 11 (13) | 7 (12) | 21 (14) | 11 (15) |
| **Headache**  mean (SD) | Pain intensity (VAS) | 2.75 (2.74) | 1.69 (2.41) | 1.88 (2.96) | 1.94 (2.72) |
|  | Days over the previous month | 12 (15) | 7 (12) | 6 (12) | 5 (9) |

**Analysis of pain intensity**:

Difference of change from baseline for chest/back pain between the 2 groups (A vs B+C):

-1.96 [-4.12 ; 0.22] (mean [CI95%]) p = 0.076

Difference of change from baseline for neck pain between the 2 groups (A vs B+C):

0.81 [-1.49 ; 3.10] p = 0.485

Difference of change from baseline for headache between the 2 groups (A vs B+C):

-1.12 [-2.79 ; 0.56] p = 0.187

**Analysis of days in pain**:

Difference of change from baseline for the number of days in chest/back pain between the 2 groups (A vs B+C): -7 [-18 ; 3] p = 0.170

Difference of change from baseline for the number of days in neck pain between the 2 groups (A vs B+C): 6 [-4 ; 16] p = 0.212

Difference of change from baseline for the number of days in headache between the 2 groups (A vs B+C): -4 [-13 ; 4] p = 0.300
