# Supplementary material for: Impact of Osteopathic Treatment on Pain in Adult Patients with Cystic Fibrosis – A Pilot Randomized Controlled Study
Source: PLoS One. 2014 Jul 16;9(7):e102465. doi: 10.1371/journal.pone.0102465 (PMC4100932; doi:10.1371/journal.pone.0102465)
Supplement: Checklist S1 — CONSORT checklist. (DOC) [file pone.0102465.s006.doc]

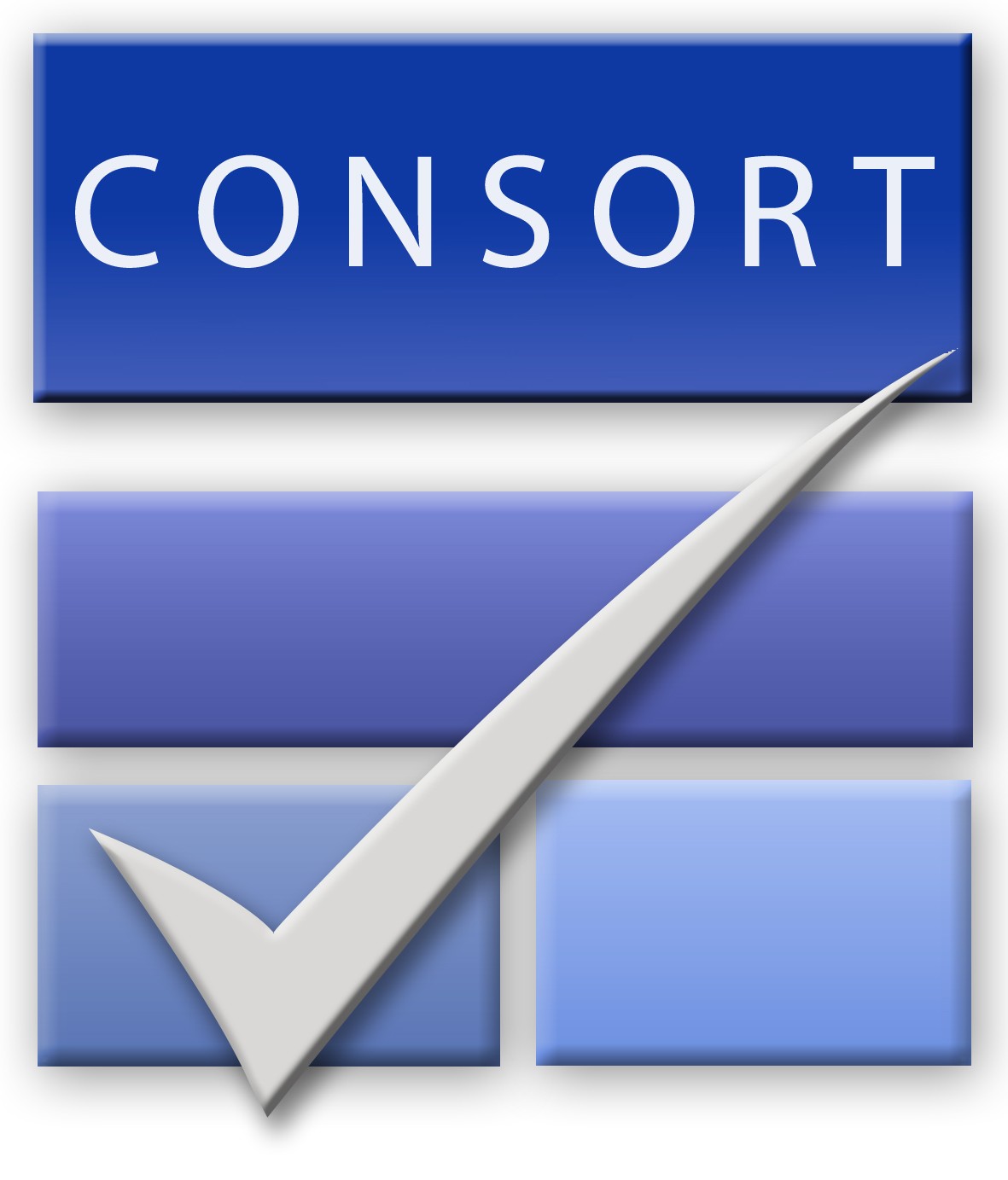
CONSORT checklist

| Section/Topic | Item No | Checklist item | Reported on | |  | |
| --- | --- | --- | --- | --- | --- | --- |
| Title and abstract | | | | |  | |
|  | 1a | Identification as a randomised trial in the title | | Title |  | |
|  | 1b | Structured summary of trial design, methods, results, and conclusions (for specific guidance see CONSORT for abstracts) | | Abstract |  | |
| Introduction | | | | |  | |
| Background and objectives | 2a | Scientific background and explanation of rationale | | Introduction first paragraph |  | |
|  | 2b | Specific objectives or hypotheses | | Introduction second paragraph |  | |
| Methods | | | | |  | |
| Trial design | 3a | Description of trial design (such as parallel, factorial) including allocation ratio | | Materials and Methods section - study design subsection |  | |
|  | 3b | Important changes to methods after trial commencement (such as eligibility criteria), with reasons | | NA |  | |
| Participants | 4a | Eligibility criteria for participants | | Materials and Methods section - setting and participants subsection |  | |
|  | 4b | Settings and locations where the data were collected | | Materials and Methods section - setting and participants subsection |  | |
| Interventions | 5 | The interventions for each group with sufficient details to allow replication, including how and when they were actually administered | | Materials and Methods section - intervention and comparator subsection - and supplementary methods | | |
| Outcomes | 6a | Completely defined pre-specified primary and secondary outcome measures, including how and when they were assessed | | Materials and Methods section - intervention and comparator subsection |  | |
|  | 6b | Any changes to trial outcomes after the trial commenced, with reasons | | NA |  | |
| Sample size | 7a | How sample size was determined | | Materials and Methods section - Sample size subsection |  | |
|  | 7b | When applicable, explanation of any interim analyses and stopping guidelines | | NA |  | |
| Randomisation: |  |  | |  |  | |
| Sequence generation | 8a | Method used to generate the random allocation sequence | | Materials and Methods section - Randomization subsection |  | |
|  | 8b | Type of randomisation; details of any restriction (such as blocking and block size) | | Materials and Methods section - Randomization subsection |  | |
| Allocation concealment mechanism | 9 | Mechanism used to implement the random allocation sequence (such as sequentially numbered containers), describing any steps taken to conceal the sequence until interventions were assigned | | Materials and Methods section - Randomization subsection | |  |
| Implementation | 10 | Who generated the random allocation sequence, who enrolled participants, and who assigned participants to interventions | | Materials and Methods section - Randomization subsection |  | |
| Blinding | 11a | If done, who was blinded after assignment to interventions (for example, participants, care providers, those assessing outcomes) and how | | Materials and Methods section - Randomization subsection |  | |
|  | 11b | If relevant, description of the similarity of interventions | | NA |  | |
| Statistical methods | 12a | Statistical methods used to compare groups for primary and secondary outcomes | | Materials and Methods section - Statistical analyses subsection |  | |
|  | 12b | Methods for additional analyses, such as subgroup analyses and adjusted analyses | | Materials and Methods section - Statistical analyses subsection |  | |
| Results | | | | |  | |
| Participant flow (a diagram is strongly recommended) | 13a | For each group, the numbers of participants who were randomly assigned, received intended treatment, and were analysed for the primary outcome | | Results section first paragraph and Figure 1 |  | |
|  | 13b | For each group, losses and exclusions after randomisation, together with reasons | | Results section first paragraph and Figure 1 |  | |
| Recruitment | 14a | Dates defining the periods of recruitment and follow-up | | Results section first paragraph |  | |
|  | 14b | Why the trial ended or was stopped | | NA |  | |
| Baseline data | 15 | A table showing baseline demographic and clinical characteristics for each group | | Table 1 |  | |
| Numbers analysed | 16 | For each group, number of participants (denominator) included in each analysis and whether the analysis was by original assigned groups | | Results section |  | |
| Outcomes and estimation | 17a | For each primary and secondary outcome, results for each group, and the estimated effect size and its precision (such as 95% confidence interval) | | Results section - Tables 2, 3, 4 and Figures 2,3,4 |  | |
|  | 17b | For binary outcomes, presentation of both absolute and relative effect sizes is recommended | | NA |  | |
| Ancillary analyses | 18 | Results of any other analyses performed, including subgroup analyses and adjusted analyses, distinguishing pre-specified from exploratory | | Results section - Table 2 and Figure 3 |  | |
| Harms | 19 | All important harms or unintended effects in each group (for specific guidance see CONSORT for harms) | | Results section - Last but one paragraph |  | |
| Discussion | | | | |  | |
| Limitations | 20 | Trial limitations, addressing sources of potential bias, imprecision, and, if relevant, multiplicity of analyses | | Discussion section - First and last paragraphs |  | |
| Generalisability | 21 | Generalisability (external validity, applicability) of the trial findings | | Discussion section - third and last paragraphs |  | |
| Interpretation | 22 | Interpretation consistent with results, balancing benefits and harms, and considering other relevant evidence | | Discussion section - fourth to sixth paragraphs |  | |
| Other information | | | |  |  | |
| Registration | 23 | Registration number and name of trial registry | | Abstract and Material and methods section - ethic statement subsection |  | |
| Protocol | 24 | Where the full trial protocol can be accessed, if available | | Sent with this article |  | |
| Funding | 25 | Sources of funding and other support (such as supply of drugs), role of funders | | Indicated to the Editor |  | |

*We strongly recommend reading this statement in conjunction with the CONSORT 2010 Explanation and Elaboration for important clarifications on all the items. If relevant, we also recommend reading CONSORT extensions for cluster randomised trials, non-inferiority and equivalence trials, non-pharmacological treatments, herbal interventions, and pragmatic trials. Additional extensions are forthcoming: for those and for up to date references relevant to this checklist, see [www.consort-statement.org](http://www.consort-statement.org/).
