# Supplementary material for: Impact of Osteopathic Treatment on Pain in Adult Patients with Cystic Fibrosis – A Pilot Randomized Controlled Study
Source: PLoS One. 2014 Jul 16;9(7):e102465. doi: 10.1371/journal.pone.0102465 (PMC4100932; doi:10.1371/journal.pone.0102465)
Supplement: Protocol S1 — Trial protocol. (DOCX) [file pone.0102465.s007.docx]

**Trial Protocol**

| **MUCOSTEO**  **OSTEOPATHIC APPROACH IN CYSTIC FIBROSIS**  IMPACT OF OSTEOPATHIC TREATMENT ON PAIN OF ADULT PATIENTS WITH CYSTIC FIBROSIS - A PILOT STUDY |
| --- |

**P080701**

| PRINCIPAL INVESTIGATOR | **Dr Dominique Hubert**  Hôpital Cochin APHP  Service de Pneumologie - CRCM  27 rue du Faubourg Saint-Jacques  75679 Paris Cedex 14 | | Tel : +33 1.58.41.23.78  / +33 6.07.40.41.41  Fax : 01.58.41.23.62  e-mail : [dominique.hubert@cch.aphp.fr](mailto:dominique.hubert@cch.aphp.fr) |
| --- | --- | --- | --- |
| SPONSOR | **Assistance Publique – Hôpitaux de Paris**  **Département de la Recherche Clinique & du Développement**  Hôpital Saint Louis  75010 PARIS | | Project manager : Yannick Vacher  Tel: +33 1.44.84.17.30  Fax : +33 1.44.84.17.99  e-mail: [yannick.vacher@sls.aphp.fr](mailto:yannick.vacher@sls.aphp.fr) |
| CLINICAL RESEARCH UNIT | **Unité de recherche clinique Paris Centre**  Hôpital Cochin - PavillonTarnier  89 rue d'Assas  75006 Paris  Coordinator : **Pr J. M. TRELUYER** | | Project manager**:** Raphaël Serreau  Tel: +33 1.58.41.11.80  Fax: +33 1.58.41.11.83  e-mail : raphael.serreau@cch.aphp.fr |
| STATISTICAL ANLYSIS | **Pr Isabelle Boutron**  Clinical Epidemiology Unit  INSERM U738  1, Place du Parvis Notre-Dame  75181 Paris Cedex 4 | | Tel : +33 1.44.07.39.75  Fax : +33 1.42.34.87.90  e-mail : isabelle.boutron@htd.aphp.fr |
|  | |  |  |
|  |  |  |  |

**Investigators**

| **Principal Investigator** | **Dr Dominique HUBERT**  Hôpital Cochin  CRCM  27 Rue du faubourg Saint-Jacques  75014 Paris | Tel : +33 1.58.41.23.78  Fax : +33 1.58.41.23.62  Courriel : dominique.hubert@cch.ahp.fr |
| --- | --- | --- |
| **Scientific Warrant** | **Mr Rafael Zegarra-Parodi**  A.T. Still Research Institute,  Kirksville, MO (USA) | Tel : +33 6.62.61.05.38  e-mail : rzegarraparodi@gmail.com |
| **Statistician** | **Pr Isabelle Boutron**  Centre d’Epidémiologie Clinique  INSERM U738  1, Place du Parvis Notre-Dame  75181 Paris Cedex 4 | Tel : +33 1.44.07.39.75  Fax : +33 1.42.34.87.90  E mail : isabelle.boutron@htd.aphp.fr |
| **Co-Investigator** | **Dr Dominique Grenet**  CRCM  Hôpital Foch  40 rue Worth  92150 Suresnes | Tel : +33 1.46.25.25.82  E mail : d.grenet@hopital-foch.org |
| **Osteopathic Practitioner** | **Lucile Soubeiran**  Clinical Research Unit  Hôpital Cochin  Site Tarnier  89 rue d’Assas  75006 Paris | Tel : +33 6 50 01 97 62  E mail: lucile.soubeiran@gmail.com |
| **Study coordination** | Unité de Recherche Clinique Paris-Centre  **Pr Jean-Marc Treluyer**  **Project Manager : Raphaël Serreau**  Hôpital Cochin  Site Tarnier  89 rue d’Assas  75006 Paris | Tel : +33 1 58 41 11 80  Fax : +33 1 58 41 11 83  E mail : raphael.serreauurc@gmail.com |

# 1. Objectives of the study

### 1.1 Primary objective

The primary objective is to assess the impact of osteopathic treatment on back-chest pain in adult patients with cystic fibrosis.

### 1.2 Secondary objectives

The secondary objectives are to assess the impact of osteopathic treatment:
• neck pain
• headache
• quality of life.

## 2. Trial Design

MUCOSTEO is a randomized controlled multicenter study with three parallel arms of a total duration of 24 months (duration of participation: 6 months).

Eligible patients for this study are patients with cystic fibrosis and with chest, neck and / or back pain. After obtaining consent, patients will be randomized into 3 groups: a group receiving a general osteopathic treatment, a group receiving a specific osteopathic treatment (sham treatment) and an untreated group.

The treatment allocation will be done through a centralized randomization at URC (Clinical trial Unit), from a list of block randomization. Patient and physician investigator will be unaware of the allocation of patients in groups 1 and 2. Only the osteopathic practitioner will know the result of randomization.

After the initial visit during which randomization occurs, patients randomized in groups 1 and 2 will be monitored every month until M6 by the osteopathic practitioner. Patients in all three groups will be reviewed at M3 and M6 to collect evaluation criteria including assessment of pain, quality of life questionnaires and osteopathic assessment.

|  | **M0** | **M1** | **M2** | **M3** | **M4** | **M5** | **M6** |
| --- | --- | --- | --- | --- | --- | --- | --- |
| Inclusion /signature of consent | X |  |  |  |  |  |  |
| Randomization | X |  |  |  |  |  |  |
| Evaluation of pain | X |  |  | X |  |  | X |
| Quality of life questionnaire | X |  |  | X |  |  | X |
| Clinical exam | X |  |  | X |  |  | X |
| PFT (FEV1) | X |  |  | X |  |  | X |
| concomitant therapies (including analgesics) | X |  |  | X |  |  | X |
| Osteopathic evaluation | X |  |  | X |  |  | X |
| General (group 1) or specific (group 2) osteopathic treatment | X | X | X | X | X | X |  |
| Adverse events |  | X | X | X | X | X | X |

## 3. Study Population

**Inclusion criteria:**

- Diagnosis of cystic fibrosis (positive sweat test and/or two CFTR disease-causing mutations)
- Age older than 18
- Patient with chest, neck or back pain (VAS> 2 or use of analgesics in the month before enrollment)
- Signature of consent
- Health insurance

**Non inclusion criteria :**

- Regular treatment of the patient by an osteopathic practitioner in the previous 3 months
- Patient on a lung transplant waiting list
- History of lung transplantation
- Pregnant woman
- Difficulties in understanding not allowing care
- Patient participation in another therapeutic clinical trial protocol

##

## 4. Data collection

**At enrollment:**

*For all patients*

- Patient's identification (first letter of first name and first letter of name)
- Sex
- Date of signature of consent
- Age at enrollment
- Randomization arm
- Verification of inclusion and non inclusion criteria
- Collecting medical history
- Clinical exam
  - Date of exam
  - Age
  - Height
  - Weight
  - Pulmonary Function tests (FVC, FEV1)
  - Pulmonary infections
  - Clinical observation (Respiratory tract, digestive tract, cardio-vascular system, neurological system, skin condition, general condition)
- Assessment of pain (mean VAS and number of days in the previous month):) for :
  - neck and trapezus pain
  - chest and back pain
  - Headache
- Other pain (location, VAS, start date, end date, medication)
- Taking analgesics, NSAIDs, muscle relaxants or antidepressants during the previous month (name, dose, route, start date, end date, note)
- Osteopathic assessment (assessed area, severity, major or specific somatic dysfunction)
- Self-administered questionnaire of quality of life, specific for cystic fibrosis
  - physical functioning
  - energy/well-being
  - body image
  - respiratory symptoms

**Visits at M1 and M2**

*For patients in groups 1 and 2 (groups with osteopathic treatment)*

- Date of visit
- Monitoring patient notebook
- Reporting adverse events
- Painful episodes over the last month (location, VAS, start date, end date , medication)
- Taking analgesics, NSAIDs , muscle relaxants or antidepressants over the last month (dosage, route , start date, end date , indication)

**Visit at M3**

*For all patients*

- Clinical examination
  - Date of examination
  - Age
  - Height
  - weight
  - Pulmonary function test (FVC, FEV1)
  - Pulmonary infections
  - clinical examination (respiratory, digestive, cardiovascular, neurological, skin condition , general condition)
- Assessment of pain (mean VAS and number of days in the previous month) for :
  - Neck and trapezes pain
  - Chest and back pain
  - Headache
- Other pain (location, VAS, start date, end date, medication)
- Taking analgesics, NSAIDs , muscle relaxants or antidepressants over the last month (dosage, route , start date, end date , indication)
- Osteopathic results (assessed area, severity, major or specific somatic dysfunction)
- Self-administered questionnaire of quality of life, specific for cystic fibrosis
  - physical functioning
  - energy/well-being
  - body image
  - respiratory symptoms

**Visits at M4 and M5**

*For patients in groups 1 and 2 (groups with osteopathic treatment)*

Same data as for visits 1 and 2

**Visit at M6**

*For all patients*

- Same data as for visit 3
- Satisfaction questionnaire

**5. Declaration of adverse events and severe adverse events**

### Non serious adverse events:

Any adverse event observed during and following the research will be reported in the CRF in the section provided for this purpose. The event may be a symptom, a diagnosis or the result of an additional exam considered significant. All clinical and paraclinical evidence that best describe the corresponding event should be reported.

### Serious adverse events (SAE):

The investigator should inform in real time, the sponsor AP-HP, of any serious adverse event.

The investigator completes the form of SAE reporting of the CRF and sends it by fax (01 44 84 17 99) to DRCD on behalf of the project manager in charge of the research, within 48 hours (if possible after a call at 01 44 84 17 23 in case of death or unexpected life-threatening event). The investigator must also inform the Paris Centre Clinical Unit Centre in charge of the research of the occurrence of SAEs.

For each serious adverse event, the investigator shall issue a medical opinion on the possible relationship between the occurrence of the event and the protocol.

Any safety data or any developments that could significantly change the assessment of the benefits and risks of the research, or that could lead to consider amendments regarding the conduct of the research, will be provided by the sponsor to the competent authorities, the IRB and the investigators of the study

# 6. Statistical analysis plan

## 6.1. Definition of endpoints

### 6.1.1 Primary endpoint

For the primary analysis, a composite endpoint will be used. For each patient the following two parameters will be averaged:

1) The evaluation of chest and back pain on the previous month (VAS 0 to 10)

2) (The number of days in the month when the patient experienced chest back pain / Total days of the previous month) *10 (score from 0 to 10)

### 6.1.2 Secondary endpoints

Secondary endpoints are:

- The mean between (the evaluation of neck and trapezus pain on the previous month) and (the number of days in the month when the patient experienced neck and trapezus pain) / (total days of the previous month) *10.
- The mean between (the evaluation of headache on the previous month) and (the number of days in the month when the patient experienced headache) / (total days of the previous month) *10.
- For quality of life, each of the 4 studied scores (physical functioning, energy / well-being, body image and respiratory symptoms) will be standardized (values ranging from 0 to 100). The average of these four standardized scores will be used as an endpoint.

## 6.2. Number of subjects required

Total number of patients planned: n = 32

Number of patients receiving general osteopathic treatment (group 1): n=16

Number of patients receiving specific osteopathic treatment (sham treatment)(group 2 ): n=8

Number of patients for the untreated control group (group 3): n = 8

We recall that this is a pilot study, in the case of a rare disease, "justifying" the small number of patients planned. However, the number of subjects should make it possible to have 80% power to demonstrate a difference of about one standard deviation of the mean VAS for pain in the group treated by general osteopathy versus the two other groups (treated by specific osteopathy or untreated (performing bilateral test with a significance level of 5%).

## 6.3. Statistical analysis

### 6.3.1 Population analysis

Only the intent-to-treat (ITT) population will be analyzed. The ITT population was defined as all randomized subjects. They will be analyzed in the group they were randomized regardless of the treatment actually received, regardless of their fate in the study.

**6.3.2 Descriptive statistics**

For the data collected at the inclusion visit, a descriptive analysis will be performed per group. Qualitative variables are described by their number, percentage and missing data by response category. Quantitative variables are described by their value, median and interquartile range [25th percentile, 75th percentile].

For osteopathic assessment, prevalence, severity index and the "burden" will be calculated for each anatomical region. The prevalence is calculated for each degree of severity (mild, moderate, severe) of the studied area and globally.

Example of descriptive analysis for an osteopathic assessment (n=1331 patients)^[[1]](#footnote-1)^ :

|  | Prevalence, n (%) | | | |  |  |
| --- | --- | --- | --- | --- | --- | --- |
| Anatomic area | Mild | Moderate | Severe | Total | Severity index | Burden |
| Head | 20 (49) | 19 (46) | 2 (5) | 41 (3.1) | 1.56^a^ | 4.8^b^ |

^a^(20x1 + 19x2 + 2x3)/41 = 1.56, ^b^3.1x1.56 = 4.8

No statistical test comparing groups from randomization will be performed at baseline.

### 6.3.3 Main analysis

The comparison will be made between the "general osteopathic treatment" group and the combination of the two "specific osteopathic (sham) treatment" and "no treatment" groups.

The final composite endpoint (at M6) for chest and back pain will be compared using a linear mixed model with random intercept at the individual patient level in the context of Mixed Model for Repeated Measurements (MMR). This type of model can take into account the correlation of data for the same patient. Bilateral tests will be made at a 5% level.

### 6.3.4 Secondary analyses

For comparison, the results for the primary endpoint will also be given for the comparison of the "general osteopathic treatment group" vs. the "specific osteopathic treatment", and the "general osteopathic treatment group" vs. the "no treatment group". Bilateral tests will be realized at a 1.33% level (instead of 5%) in order to take into account the multiplicity induced by these two sensitivity analyses.

The final composite criteria (at M6) for neck and trapezius pain, headache and the 4 studied scores of quality of life (physical functioning, energy/well-being, body image, respiratory symptoms) will be analyzed using the same strategy as for the primary endpoint.

### 6.3.5 Handling missing data

No imputation strategy will be realized: the analysis being an analysis by mixed models, the parameters are estimated without bias in case of missing data under the assumption that these data are missing randomly.

### 6.3.6 Software used

Statistical analysis will be carried out by the Clinical Epidemiology Unit by Elodie Perrodeau under the responsibility of Dr Isabelle Boutron using the SAS 9.2 and/or R.2.13 software.

1. Licciardone et al, Osteopathic manipulative treatment of somatic dysfunction among patients in the family practice clinic settings : a retrospective analysis, JAOA, Vol 105, No12, Dec 2005, 537-543. [↑](#footnote-ref-1)
