# Supplementary material for: Impact of Osteopathic Treatment on Pain in Adult Patients with Cystic Fibrosis – A Pilot Randomized Controlled Study
Source: PLoS One. 2014 Jul 16;9(7):e102465. doi: 10.1371/journal.pone.0102465 (PMC4100932; doi:10.1371/journal.pone.0102465)
Supplement: Methods S1 — Supplementary methods. (DOCX) [file pone.0102465.s008.docx]

**Supplementary Methods**

**Diagnostic procedures**

All patients underwent a comprehensive osteopathic examination at the base-line visit (M0), at 3 months (M3) and at 6 months (M6). Osteopathic diagnostic procedures were the same for the three groups and categorized into cranial, musculoskeletal and visceral tests [1S, 2S]. We used the clinical decision criteria for the presence and the severity of somatic dysfunctions as described in the Outpatient Osteopathic SOAP Note Form, a subjective and objective assessment tool designed by the American Academy of Osteopathy (Table S1) [1S]. The four clinical signs associated with somatic dysfunctions were: sensitivity/tenderness to palpation, anatomical landmarks asymmetry, restriction of passive mobility and tissue texture changes. Somatic dysfunction severity was rated from 0 (absence) to 3 (severe) depending on the presence of these clinical signs for each of the 14 areas of the body.

Patients were evaluated in different positions: lateral recumbent for the lumbar spine, seated for the thoracic spine and ribs, and supine for the other areas of the body.

**Intervention**

Patients with usual care did not receive osteopathic manipulative treatment (OMT). All the other patients received six sessions of OMT, every month from M0 to M5: either “OMT”, the experimental treatment, or “sham OMT”, the sham treatment.

***Osteopathic manipulative treatment***

A standardized treatment plan was designed based on the diagnosed somatic dysfunctions and their potential influence on related structures according to their neurophysiological relationships [3S].

OMT was applied on: (1) somatic dysfunctions related to chest and back pain as described by the patient (i.e. thoracic and rib somatic dysfunctions), (2) somatic dysfunctions most frequently affected by cystic fibrosis (thoraco-pulmonary area and respiratory muscles) [4S] and (3) severe somatic dysfunctions (rated 3) as diagnosed by the practitioner.

Each visit included 6 sequences always performed in the same order. Techniques used were specific depending on the diagnostic procedures used for cranial, musculoskeletal and visceral areas [3S]. The number of techniques for each of the areas ranged from 3 to 7 techniques and each one lasted about 2 minutes (Table S2). The treatment itself lasted about 20 minutes (Table S3). If patient’s positioning was painful or not comfortable, the same OMT was applied in another position (Table S4).

***Sham osteopathic manipulative treatment***

To ensure that manual contact time was similar (20 minutes) in the experimental and sham treatment groups, patients in the sham treatment group received light touch [5S-6S] at the skull and at the sacrum regardless of the cranial somatic dysfunctions found (Table S3).

**Supplementary References**

1S. American Academy of Osteopathy. Outpatient Osteopathic SOAP Note Form Series and Usage Guide.2^nd^ ed. Indianapolis, Ind: American Academy of Osteopathy, 2002. Available at: <http://www.academyofosteopathy.org/>

2S. American Association of Colleges of Osteopathic Medicine. Authorized Osteopathic Thesaurus. Available at: <http://www.aacom.org/resources/bookstore/thesaurus/Pages/default.aspx>. Accessed July 14, 2012.

3S. World Health Organization. Benchmarks for training in traditional / complementary and alternative medicine: benchmarks for training in osteopathy. WHO Press, Geneva, Switzerland. 2010. Available at: <http://www.who.int/medicines/areas/traditional/BenchmarksforTraininginOsteopathy.pdf>. Accessed on July 13, 2013.

4S. Soubeiran L, Hubert D, Serreau R, Desmazes_Dufeu N, Zegarra-Parodi R. Prevalence of somatic dysfunctions in adult patients with cystic fibrosis – a pilot study. *J Phys Ther.* 2011; 4:18-31.

5S. Noll DR, Degenhardt BF, Morley TF, et al. Efficacy of osteopathic manipulation as an adjunctive treatment for hospitalized patients with pneumonia: a randomized controlled trial. *Osteopath Med Prim Care*. 2010 Mar 19; 4:2. doi: 10.1186/1750-4732-4-2.

6S. Noll RD, Degenhardt BF, Fossum C, Hensel K. Clinical and research protocol for osteopathic manipulative treatment of elderly patients with pneumonia. *J Am Osteopath Assoc*. 2008; 108(9):508-516.
